# Causes of end stage renal failure among haemodialysis patients in Khartoum State/Sudan

**DOI:** 10.1186/s13104-015-1509-x

**Published:** 2015-09-29

**Authors:** Amin S. I. Banaga, Elaf B. Mohammed, Rania M. Siddig, Diana E. Salama, Sara B. Elbashir, Mohamed O. Khojali, Rasha A. Babiker, Khalifa Elmusharaf, Mamoun M. Homeida

**Affiliations:** Haemodialysis Unit, Department of Medicine and Nephrology, University of Medical Sciences and Technology, Academy Charity Teaching Hospital, P.O. Box. 12810, Khartoum, Sudan; Department of Nephrology, Academy Charity Teaching Hospital, P.O. Box. 12810, Khartoum, Sudan; Department of Basic Sciences, Faculty of Medicine, University of Medical Sciences and Technology, P.O. Box. 12810, Khartoum, Sudan; Epidemiology and Public Health, Faculty of Medicine, Royal College of Surgeon in Ireland RCSI Bahrain, P.O. Box. 15503, Adliya, Bahrain; Department of Medicine, Faculty of Medicine, University of Medical Sciences and Technology, P.O. Box. 12810, Khartoum, Sudan

**Keywords:** Sudan, Khartoum, End stage renal failure, Hypertension, Diabetes, Glomerulonephritis

## Abstract

**Background:**

End stage renal failure (ESRF) has become a major health problem in Sub Saharan Africa (SSA). There were limited data about causes of ESRF in the Sudan.

**Methods:**

This is a cross sectional hospital based descriptive study. The subjects of the study are ESRF adults’ patients on regular haemodialysis treatment in 15 haemdoialysis centres in Khartoum State—Sudan. Clinical and epidemiological data were obtained from 1583 patients. The medical files of each patient were reviewed to identify the cause of ESRF. Concerning the causes of ESRF, diabetes was diagnosed based on the past medical history and result of the glucose tolerance test, hypertension was diagnosed based on past history of hypertension based on blood pressure of more than 140/90 mmHg, glomerulonephritis was diagnosed based on results of previous kidney biopsies and on clinical grounds, polycystic kidney disease and obstructive uropathy were diagnosed based on abdominal ultrasound and other imaging modalities, sickle cell anaemia was diagnosed based on the result of haemoglobin electrophoresis, systemic lupus erythematosus was diagnosed based on the clinical criteria in addition to lab results of auto antibodies, and analgesic nephropathy was diagnosed based on past medical history of chronic analgesic drugs usage with no other identifiable risk factors. We included all ESRF patients on regular haemodialysis treatment. We excluded ESRF patients less than 18 years old.

**Results:**

The results showed that the mean age of ESRF Patients was 49 ± 15.8 (years) and 63.4 % were male and 76.3 % were unemployed. The mean duration of haemodialysis is 4.38 ± 4.24 (years). The most common cause of ESRF in our patients was hypertension (34.6 %) followed by chronic glomerulonephritis (17.6 %), diabetes mellitus (12.8 %), obstructive uropathy (9.6 %), autosomal dominant poly cystic kidney disease (ADPKD) (4.7 %), chronic pyelonephritis (4.6 %), analgesic nephropathy (3.5 %). However in (10.7 %) no cause was found. In patient aged less than 40 years old the leading cause of ESRF was glomerulonephritis (29.3 %) followed by hypertension (25 %). In patient aged between 40 to 60 years old the leading cause of ESRF was hypertension (38.5 %) followed by diabetes mellitus (14 %). In patient aged older than 60 years the leading cause of ESRF was hypertension (38.4 %) followed by diabetes mellitus (23.3 %).

**Conclusions:**

ESRF in Sudan affects the economically productive age group; unemployment rate among ESRF patients is high. The study showed that hypertension is a leading cause of ESRF in Sudan followed by chronic glomerulonephritis. Hypertension and diabetes mellitus are the leading causes of ESRF among patients over 40 years old.

## Background

End stage renal failure (ESRF) has become a major health problem in Sub Saharan Africa (SSA). There are limited data on the prevalence and incidence of ESRF in SSA due to lack of renal registries. Several studies pointed out to the magnitude of the problem in SSA. In Nigeria a study reported an increase of hospital admissions because of ESRF from 6 to 16 % between the years 1989 and 2003 [1]. In Senegal only 8.23 % of ESRF patients receive renal replacement therapy (RRT) [2]. In Ghana, a study pointed out that 5 % of total hospital admissions had renal disease of whom 27.1 % died, usually of ESRF [3]. Hypertension is a leading cause of ESRF in Senegal and Ghana [4, 5] while chronic glomerulonephritis is the leading cause in South Africa and Ivory Coast [6, 7].

In Sudan, the estimated incidence of new cases of ESRF patients is 70–140 per million inhabitants/year [8]. There were limited data about causes of ESRF in Sudan. A small study conducted in Sudan among 61 patients in 1987 reported that the causes of chronic kidney disease (CKD) are chronic glomerulonephritis, obstructive nephropathy, hypertension and diabetes mellitus in that order [9]. Furthermore in study conducted among 100 Sudanese patients, chronic glomerulonephritis was found to be the leading cause of ESRF [10]. Other study conducted in central Sudan in 2009 among 224 ESRF patients found that hypertension (14.3 %) is a leading cause of ESRF followed by obstructive uropathy (11.6 %), glomerulonephritis (9.8 %), diabetes mellitus (8 %), however in (53.57 %) no cause was found [11]. The aim of this study is to update and outline the causes of ESRF in Sudan.

## Methods

### Study population and data collection

This study is a cross sectional hospital based descriptive study. The subjects are ESRF adults’ patients on regular haemodialysis treatment in 15 haemdoialysis centres in Khartoum/Sudan. All patients on regular haemodialysis were studied in November 2014 and interviewed by questionnaire focusing on personal and clinical data including (age, address, origin, employment, duration of dialysis, cause of ESRF). The medical files of each patient were reviewed to identify the cause of ESRF.

Concerning the causes of ESRF, diabetes was diagnosed based on the past medical history and result of the glucose tolerance test, hypertension was diagnosed based on past history of hypertension based on blood pressure of more than 140/90 mmHg, glomerulonephritis was diagnosed based on results of previous kidney biopsies and on clinical grounds, polycystic kidney disease and obstructive uropathy were diagnosed based on abdominal ultrasound and other imaging modalities, sickle cell anaemia was diagnosed based on the result of haemoglobin electrophoresis, systemic lupus erythematosus was diagnosed based on the clinical criteria in addition to lab results of auto antibodies, and analgesic nephropathy was diagnosed based on past medical history of chronic analgesic drugs usage with no other identifiable risk factors.

The research was in compliance of declaration of Helsinki and approved by ethics and research committees in the Ministry of Health/Sudan and local hospitals. An informed consent was obtained from each patient participated in the study.

### Inclusion and exclusion criteria

We included all haemodialysis patients in 15 haemodialysis centres in Khartoum/Sudan between (1/11/2014 and 1/12/2014). We excluded haemodialysis patients less than 18 years old.

### Statistical analysis

Data were analyzed using SPSS 21; results were presented in number, percent, mean and standard deviation.

## Results

A total of 1583 ESRF patients participated in the study. The characteristics of the study population are shown in Table 1. The results showed that the mean age of ESRF Patients was 49 ± 15.8 (years) and 63.4 % were male and 76.3 % were unemployed. The mean duration of haemodialysis is 4.38 ± 4.24 (years).

**Table 1:** The characteristics of the study population

Age (years)^b^	49 ± 15.8
Gender^a^	
Male	1004 (63.4 %)
Female	579 (36.6 %)
Occupation^a^	
Unemployed	1208 (76.3 %)
Non professional	238 (15.0 %)
Professional	137 (8.4 %)
Origin^a^	
Northern Sudan	438 (27.7 %)
Central Sudan	417 (26.3 %)
Western Sudan (Kordofan)	270 (17.1 %)
Western Sudan (Darfour)	144 (9.1 %)
Eastern Sudan	53 (3.3 %)
Khartoum	261 (16.5 %)
Address^a^	
Khartoum	624 (39.4 %)
Omdurman	547 (34.6 %)
Khartoum North	230 (14.5 %)
East Nile	147 (9.3 %)
Outside Khartoum	35 (2.2 %)
Duration of haemodialysis (years)^b^	4.38 ± 4.24

^a^Number (percentage)

^b^Mean ± SD

The aetiology of ESRF is shown in Table 2. The most common cause of ESRF in our patients was hypertension (34.6 %) followed by chronic glomerulonephritis (17.6 %), diabetes mellitus (12.8 %), obstructive uropathy (9.6 %), autosomal dominant poly cystic kidney disease (ADPKD) (4.7 %), chronic pyelonephritis (4.6 %), analgesic nephropathy (3.5 %). However in (10.7 %) no cause was found.

**Table 2:** Causes of end stage renal failure among study population

Hypertensive nephropathy	547 (34.6 %)
Glomerulonephritis	278 (17.6 %)
Diabetic nephropathy	203 (12.8 %)
Obstructive uropathy	152 (9.6 %)
Polycystic kidney disease	74 (4.7 %)
Chronic pyelonephritis	73 (4.6 %)
Analgesic nephropathy	56 (3.5 %)
Congenital and hereditary diseases	15 (0.9 %)
Systemic lupus erytherematosus	9 (0.6 %)
Sickle cell anaemia	7(0.4 %)
Unknown	169 (10.7 %)
Total	1583 (100 %)

Data are numbers (percent)

In patient aged less than 40 years old the leading cause of ESRF was glomerulonephritis (29.3 %) followed by hypertension (25 %). In patient aged between 40 to 60 years old the leading cause of ESRF was hypertension (38.5 %) followed by diabetes mellitus (14 %). In patient aged older than 60 years the leading cause of ESRF was hypertension (38.4 %) followed by diabetes mellitus (23.3 %). The study showed significant relation between age and some aetiologies of ESRF, glomerulonephritis, chronic pyelonephritis, sickle cell anaemia and congenital disease are tend to cause ESRF in younger patients while diabetes mellitus, hypertension and polycystic kidney disease are significantly cause ESRF in older patients (Table 3).

**Table 3:** Causes of end stage renal failure according to age

Causes of end stage renal failure	<40 years (n = 463)		40–60 years (n = 716)		>60 years (n = 404)		P value
	Count	%	Count	%	Count	%	
Glomerulonephritis	136	29.4	100	14.0	42	10.4	0.000
Hypertensive nephropathy	116	25.1	276	38.5	155	38.4	0.000
Obstructive uropathy	42	9.1	77	10.8	33	8.2	0.333
Chronic pyelonephritis	39	8.4	20	2.8	14	3.5	0.000
Analgesic nephropathy	19	4.1	26	3.6	11	2.7	0.538
Diabetic nephropathy	10	2.2	99	13.8	94	23.3	0.000
Congenital and hereditary diseases	9	1.9	5	0.7	1	0.2	0.024
Polycystic kidney disease	8	1.7	50	7	16	4	0.0001
Sickle cell anaemia	6	1.3	0	0	1	0.2	0.004
Systemic lupus erythematosus	4	0.9	4	0.6	1	0.2	0.484
Unknown	74	16	59	8.2	36	8.9	0.0001

Data are numbers (percent)

We found no regional differences in causes of ESRF in Sudan (Table 4).

**Table 4:** Causes of end stage renal failure according to region

Causes	Region						Total
	North Sudan	Central Sudan	Kordofan (West Sudan)	Darfour (West Sudan)	Eastern Sudan	Khartoum	
Hypertension	146	132	112	43	16	98	547
	33.3 %	31.7 %	41.5 %	29.9 %	30.2 %	37.5 %	34.6 %
Diabetes mellitus	74	53	23	11	6	36	203
	16.9 %	12.7 %	8.5 %	7.6 %	11.3 %	13.8 %	12.8 %
Glomerulonephritis	69	73	48	37	7	44	278
	15.8 %	17.5 %	17.8 %	25.7 %	13.2 %	16.9 %	17.6 %
Obstructive uropathy	41	49	21	18	8	15	152
	9.4 %	11.8 %	7.8 %	12.5 %	15.1 %	5.7 %	9.6 %
Polycystic kidney disease	27	17	7	4	4	15	74
	6.2 %	4.1 %	2.6 %	2.8 %	7.5 %	5.7 %	4.7 %
Chronic pyelonephritis	20	15	13	5	5	15	73
	4.6 %	3.6 %	4.8 %	3.5 %	9.4 %	5.7 %	4.6 %
Analgesic nephropathy	8	18	12	6	1	11	56
	1.8 %	4.3 %	4.4 %	4.2 %	1.9 %	4.2 %	3.5 %
Systemic lupus erythrematosus	5	1	2	0	1	0	9
	1.1 %	0.2 %	0.7 %	0.0 %	1.9 %	0.0 %	0.6 %
Sickle cell anaemia	1	1	3	0	1	1	7
	0.2 %	0.2 %	1.1 %	0.0 %	1.9 %	0.4 %	0.4 %
Congenital and hereditary diseases	10	4	0	0	0	1	15
	2.3 %	1.0 %	0.0 %	0.0 %	0.0 %	0.4 %	0.9 %
Unknown	37	54	29	20	4	25	169
	8.4 %	12.9 %	10.7 %	13.9 %	7.5 %	9.6 %	10.7 %
Total	438 (100 %)	417 (100 %)	270 (100 %)	144 (100 %)	53 (100 %)	261 (100 %)	1583 (100 %)

## Discussion

### Characteristics of study population

CKD is at least 3–4 times more frequent in Africa than in developed countries [12]. Haemodialysis treatment in Sudan is free and paid by the government. There are limited data on the prevalence and causes of ESRF in Sudan apart from few studies. This study included a large population of ESRF patients.

CKD in SSA tends to affect relatively younger individuals [13]. Our result showed that the mean age of ESRF patients is 49 ± 15.8 (years) and 47.9 % of ESRF patients are below an age of 50 years. This indicates that ESRF in Sudan affects the economically productive age group, unlike the situation in many developed countries were the mean age of ESRF patients is generally over 60 years [14].

In this study males constitute 63 % of ESRF patients receiving haemodialysis treatment; this is similar to many of other study conducted in Africa, in Ethiopia males constitute 61.5 % of ESRF patients receiving dialysis [15], in Ivory Coast males constitute 61 % of patients [16].

Several studies pointed out an increase rate of unemployment among haemodialysis patients [17, 18]. In our study 76.3 % of ESRF patients receiving haemodialysis treatment were unemployed; this reflect the financial burden on families of patients on haemodialysis especially in an African country like Sudan.

### Causes of ESRF

In the current study hypertension is a leading cause of ESRF as it is a leading cause in many of SSA countries [4, 5]. Other study conducted in central Sudan in 2009 among 224 patients found that hypertension is a leading cause of ESRF [11]. Hypertension is considered to be a common health problem in SSA. 33 % of the general population in Malawi were found to be hypertensive [19]. In Uganda the prevalence of hypertension was 22.1 % in men and 20.5 % in women [20]. In Nigeria, 20.8 % of general population found to be hypertensive [21]. Hypertension in SSA has been mounting over the past few decades. A meta analysis of studies conducted between 2000 and 2013 in SSA found that only 18 % of individuals with hypertension were receiving treatment and only 7 % had controlled blood pressure [22]. In other community based Sudanese survey conducted in 2012 found that the prevalence of hypertension in rural areas was 15.8 and 45 % had uncontrolled blood pressure [23].

In this study, chronic glomerulonephritis was found to be the second most frequent cause of ESRF. The previous two published studies conducted in Sudan on 1987 and 1989 found that chronic glomerulonephritis is a leading cause of ESRF [9, 10], however both studies were conducted in one centre with small study samples. Our results are relevant to many of SSA where the glomerulonephritis was found to be the second leading cause of ESRF. In Nigeria, 27.8 % of causes of ESRF is attributed to chronic glomerulonephritis [1]. In Ghana, glomerulonephritis is a second leading cause of ESRF [5]. In Senegal, 16 % of ESRF patients are due to chronic glomerulonephritis and consider to be the second leading cause of ESRF following hypertension [4]. The situation is different in South Africa where glomerulonephritis is a leading cause of ESRF [6].

Our result showed that diabetes mellitus was found to be the second leading cause of ESRF among patients over 40 years old. There are limited data on prevalence of diabetes mellitus in Sudan. A community based study conducted in Sudan in 1996 conducted among 1284 subjects found that prevalence of diabetes mellitus was 3.4 % [24]. Other study conducted in Dongola in North Sudan found that prevalence of diabetes mellitus was 8.3 % [25]. Diabetes is the leading cause of ESRF in Latin America [26] and in UK black patients [27]. In USA, the incidence of ESRF due to diabetes was 2.6 fold higher among blacks [28]. Diabetes is a second leading cause of ESRF in many of SSA like in Nigeria [29] and in Senegal [30]. The prevalence of diabetic nephropathy in SSA is estimated to be 14–16 % in South Africa, 23.8 % in Zambia, 9 % in Sudan, and 6.1 % in Ethiopia [12].

Analgesic nephropathy accounted for a significant minority of causes of ESRF in Sudan. This is because the excessive use of analgesia without doctor’s prescription. Analgesic nephropathy first attributed to the habitual use of phenacetin-containing analgesics [31]. Several studies pointed out that an excessive use of analgesic mixtures containing acetaminophen, aspirin, caffeine, or codeine also can be associated with analgesic nephropathy and eventually ESRF [32, 33]. Several studies also pointed out to the association between the use of single-ingredient analgesics containing acetaminophen or aspirin and CKD [34–36]. A Swedish study conducted in 2001 found that the regular use of acetaminophen or aspirin was associated with a risk of chronic renal failure that was 2.5 times as high as that for nonusers [37]. An American study found that patients use non steroidal anti inflammatory drugs (NSAID) in daily bases were associated with twofold increase risk for CKD [38]. NSAID have been associated with nephrotic syndrome, interstitial nephritis and ESRF [39], despite that patient with CKD should avoid the use of NSAID, still studies found that CKD awareness was not associated with reduction of NSAID usage [40].

In our study, 4.7 % of ESRF patients receiving dialysis are due to autosomal dominant polycystic kidney disease (ADPKD) and it remains the leading hereditary cause of ESRF in Sudan. Results obtained from European registries stated that ADPKD constitute about 9.8 % of ESRF patients receiving RRT [41]. There were limited data about prevalence of ADPKD in Africa. Our data is similar to other African studies. In Morocco, 6.5 % of patients on dialysis are due to ADPKD [42] similar to Libya which is 6.3 % [43]. A hospital study conducted in Senegal found that prevalence of ADPKD was one in 250 patients following in Nephrology Department [44].

Small percentage of adult ESRF patients receiving RRT in Sudan is due to sickle cell anemia. Despite sickle cell anaemia is common in Sudan however they die early. Sickle cell anaemia is associated with renal ischemia, glomerulonephritis, nephrotic syndrome and ESRF [45, 46]. There is limited data on sickle cell nephropathy in SSA. In USA, 0.11 % of ESRF patients receiving renal replacement therapy are due to sickle cell anemia and 93 % were African–Americans [47].

In our study, we couldn’t identify a known cause of ESRF in 10.7 % of patients. This is mostly because of late presentation of patients to the nephrology departments and lack of medical facilities and poor medical follow up of patients in rural areas.

The strength of this study is the inclusion of all ESRF patients receiving haemodialysis treatment in 15 haemodialysis units in Khartoum State/Sudan with a total of 1583 patients participated in the study makes our study has a large sample size, putting in mind that 60 % of ESRF patients in Sudan receive RRT in Khartoum State. Our study has limitations that need to be addressed. In our study, hypertension is a leading cause of ESRF, but in some cases it was difficult to determine is it primary hypertension or secondary to CKD itself? This is because of lack of regular medical follow up in our patients. Other point which need to be discussed is that in this study, identifications of diabetes and hypertension as an aetiology of ESRF can be overestimated, lack of kidney biopsies from these patients that needed it to make sure that there is no other cause of ESRF and make an exact determination of the aetiology of ESRF is difficult, this is because of late presentation of patients and lack of resources. Many studies conducted in developing countries used the same criteria we used to determine the aetiology of ESRF [48, 49]. Other limitation is that sometimes the medical data about the exact nature of the cause of ESRF are not available especially data regarding kidney biopsies which made the percentage of the unknown cause is slightly increased in our study. In addition, our study conducted in haemodialysis patients but there is no available data about ESRF patients not on RRT.

## Conclusions

ESRF in Sudan affects the economically productive age group; unemployment rate is high among ESRF patients. The study showed that hypertension is a leading cause of ESRF in Sudan followed by chronic glomerulonephritis. Hypertension and diabetes mellitus are the leading causes of ESRF among patients over 40 years old.

## Abbreviations

ADPKD: autosomal dominant polycystic kidney disease
CKD: chronic kidney disease
ESRF: end stage renal failure
RRT: renal replacement therapy
SSA: Sub Saharan Africa
NSAID: non steroidal anti inflammatory drugs
